# The Association between Physical Health and Retirement Planning: Findings from the Health and Retirement Study

**DOI:** 10.1093/geroni/igaf122.2764

**Published:** 2025-12-31

**Authors:** Yan-Jhu Su, Andrew Alberth, Elisabeth Stam, Alison Rataj, Shu Xu, Jeffrey Stokes

**Affiliations:** The Pennsylvania State University, University Park, Pennsylvania, United States; University of Massachusetts Boston, Boston, Massachusetts, United States; University of Massachusetts Boston, Boston, Massachusetts, United States; College of Health and Human Services, Center on Aging and Community Living, Durham, New Hampshire, United States; University of Michigan, Ann Arbor, Michigan, United States; University of Massachusetts Boston, Boston, Massachusetts, United States

## Abstract

**Objectives:**

Previous health and anticipated retirement age studies have shown inconsistent results. One explanation for such discrepancies may result from differences in subjective and objective health across demographic groups. Therefore, this study examines whether self-rated poor health (subjective) and number of chronic illnesses (objective) were uniquely associated with anticipated retirement age, and whether this association differed by race or ethnicity for adults 50 years and older in the United States.

**Methods:**

This cross-sectional study uses data from the 2018 Health and Retirement Study (HRS). The sample included adults 50 years and older who were working for pay in 2018 (N = 5,970). The main dependent variable was respondents’ predicted age of retirement and the independent variables were self-rated poor health and chronic illness. Race and ethnicity were assessed as moderators. Ordinary least squares (OLS) regression models were used.

**Results:**

The main effect of self-rated poor health on anticipated retirement age indicated less healthy people would retire sooner. However, having more chronic illnesses was not associated with predicted retirement age. Chronic illness is associated with earlier planned retirement ages among Black respondents, but racial/ethnic differences were not detected among self-rated health.

**Conclusions:**

These findings suggest older adults will remain in the workforce for a longer period of time when they are – or believe themselves to be – healthy. To support the aging workforce, enhancing chronic disease prevention efforts for Black adults should be pursued. Future research should investigate how subjective and objective health may differentially impact specific industries’ workforces.

